# Association among *H. pylori* virulence markers *dupA*, *cagA* and *vacA* in Brazilian patients

**DOI:** 10.1186/1678-9199-20-1

**Published:** 2014-01-23

**Authors:** Weendelly Nayara Pereira, Mariane Avante Ferraz, Luanna Munhoz Zabaglia, Roger William de Labio, Wilson Aparecido Orcini, João Paulo Bianchi Ximenez, Agostinho Caleman Neto, Spencer Luiz Marques Payão, Lucas Trevizani Rasmussen

**Affiliations:** 1 Sacred Heart University, Bauru, São Paulo State, Brazil; 2 Department of Genetics, Blood Bank, Marília Medical School, Marília, São Paulo State, Brazil

**Keywords:** *Helicobacter pylori*, *dupA*, *cagA*, *vacA*, Gastroduodenal diseases, PCR

## Abstract

**Background:**

Only a few *Helicobacter pylori*-infected individuals develop severe gastric diseases and virulence factors of *H. pylori* appear to be involved in such clinical outcomes. Duodenal ulcer promoting gene A (*dupA*) is a novel virulence factor of *Helicobacter pylori* that is associated with duodenal ulcer development and reduced risk for gastric carcinoma in some populations. The aims of the present study were to determine the presence of *dupA* gene and evaluate the association among *dupA* and other virulence factors including *cagA* and *vacA* in Brazilian patients. Gastric biopsies were obtained from 205 dyspeptic patients (100 children and 105 adults). DNA was extracted and analyzed for the presence of *H. pylori* and its virulence factors using the polymerase chain reaction method.

**Results:**

Patients with gastritis tested positive for *H. pylori* more frequently. The *dupA* gene was detected in 41.5% of them (85/205); *cagA* gene was found in 98 isolates (47.8%) and *vacA* genotype s1/m1 in 50.2%, s1/m2 in 8.3%, s2/m2 in 36.6%, s2/m1 in 0.5% and s1/s2/m1/m2 in 4.4%. We also verified a significant association between *cagA* and *dupA* genes [p = 0.0003, relative risk (RR) 1.73 and confidence interval [CI] = 1.3–2.3]. The genotypes s1/m1 were also associated with *dupA* gene (p = 0.0001, RR: 1.72 and CI: 1.3–2.2). The same associations were found when analyzing pediatric and adult groups of patients individually.

**Conclusion:**

Ours results suggest that *dupA* is highly frequent in Brazilian patients and is associated with *cagA* gene and *vacA* s1/m1 genotype, and it may be considered an important virulence factor in the development of gastric diseases in adults or children.

## Background

*Helicobacter pylori* is a gram-negative spiral bacterium that colonizes the human stomach. It is estimated that approximately half of the world’s population is infected with it [1,2]. In 1994, the International Agency for Research on Cancer included *H. pylori* infection in group I carcinogens. In addition, several authors report that the chronic infection may induce gastritis, peptic ulcer, gastric adenocarcinoma and gastric mucosa-associated lymphoid tissue lymphoma [3-5].

It is interesting to note that only approximately 20% of *H. pylori*-infected individuals develop severe gastric diseases, suggesting that clinical outcomes are determined by the interaction among bacterial virulence, host genetic susceptibility and environmental factors [2,6].

Virulence factors of *H. pylori* – including *vacA*, *cagA* and *babA* – play important roles in gastric diseases. The *vacA* gene is present in all *H. pylori* strains and it is composed of two main regions: the signal region (s1 or s2) and the middle region (m1 or m2), both contribute to variations in the vacuolating activity of different *H. pylori* strains. s1/m1 strains are the most cytotoxic, followed by the s1/m2 strains and the s2/m2 [7].

Moreover, *cagA* gene is present in approximately 60 to 70% of *H. pylori* strains and several epidemiological studies have revealed that its presence is correlated with a higher risk of developing peptic ulceration, gastric atrophy and gastric cancer [8,9].

Most recently, Lu *et al.*[10] described a novel virulence factor, a *virb*B4 homologue, called duodenal ulcer (DU) promoting gene (*dupA*). It is located in the “plasticity region” of *H. pylori* genome and is composed of two genes, jhp0917 and jhp0918, which form one continuous open reading frame by the insertion of a base T or C after the position 1385 in the jhp0917 3’ region [6,11].

Although the role of the *dupA* gene is unclear, Lu *et al.*[10] suggested that this gene is involved in cell division and chromosomal DNA transfer. The authors also reported that infections with *dupA*-positive strains increased the risk for duodenal ulcer, but they were protective against gastric atrophy, intestinal metaplasia and gastric cancer in the Japanese, Korean and Colombian patients.

The involvement of *H. pylori* virulence factors in gastric disease has been demonstrated around the globe. Therefore, the aim of the present study was to evaluate the presence of *cagA*, *vacA* and *dupA* genes in Brazilian patients (children and adults) and to analyze the relationship between virulence factors and gastroduodenal diseases.

## Methods

### Patients and samples

We analyzed 205 samples obtained from 100 pediatric patients (41♂/59♀ mean age 10.9 ± 3.7) and 105 adult patients (57♂/48♀ mean age 47.8 ± 14.3) with abdominal symptoms who had been submitted to upper gastrointestinal endoscopy in the Department of Gastroenterology of the Marília Medical School, Brazil. All subjects or their parents signed an informed consent form approved by the Research Ethics Committee of Sacred Heart University (process n. 068/12).

Two samples from each patient were taken endoscopically from the gastric antrum, one for genotyping and detection of *H. pylori* by polymerase chain reaction (PCR) and the second for histopathological analyses. Only *H. pylori* positive patients were included in this study.

All patients were from the same socioeconomic level and had similar cultural habits. Regarding ethnic origins, approximately 40% were Caucasian, 45% were of Amerindian origin, and 15% were of mixed origin. The ethnic origins were determined by self-report and by family geographical origin.

### DNA extraction and *H. pylori* isolation

DNA from gastric biopsies was extracted using the QIAamp® tissue kit (Qiagen, Germany) according to the manufacturer´s instructions. For detection of the *H. pylori*, PCR assays were performed using one set of oligonucleotides Hpx1/Hpx2 that amplifies a 150-bp fragment corresponding to 16S-rRNA from *H. pylori* (Table 1). In each experiment, positive (strain 26695) and negative (water) controls were included.

**Table 1:** Primers and condition of amplification used in the study

**Primers**	**Primer sequence (5′-3′)**	**Amplification condition**	**Gene**	**Reference**
*DupA*1	CGTGATCAATATGGATGCTT	35 cycles: 45 s, 94°C; 45 s, 52°C and 45 s, 72°C	*dupA*	Gomes *et al.*[6]
*DupA*2	TCTTTCTAGCTTGAGCGA			
Cag1	ATGACTAACGAAACTATTGATC	40 cycles: 1 min, 94°C; 1 min, 53°C and 1 min, 72°C	*cagA*	Rasmussen *et al.*[9]
Cag2	CAGGATTTTTGATCGCTTTATT			
Hpx1^a^	CTGGAGARACTAAGYCCTCC	40 cycles: 1 min, 94°C; 1 min, 59°C and 1 min, 72°C	16S rRNA	Scholte *et al*. [12]
Hpx2	GAGGAATACTCATTGCGAAGGCGA			
SA	ATGGAAATACAACAAACACAC	40 cycles: 45 s, 94°C; 45 s, 54°C and 45 s, 72°C	*vacA-s*	Atherton *et al*. [13]
SC^a^	CCTGARACCGTTCCTACAGC			Doorn *et al*. [14]
MA^a^	CACAGCCACTTTYAATAACGA	35 cycles at: 45 s, 94°C, 45 s, 55°C and 1 min, 72°C	*vacA-m*	Doorn *et al*. [14]
MB	CGTCAAAATAATTCCAAGGG			

•Genotype *vacA*: s1/m1 – strain 60190 of *H. pylori* (GeneBank U05676).

•Genotype *vacA*: s2/m2 – strain Tx30a of *H. pylori* (GeneBank U29401).

•^a^R = a A or G and Y = C or T.

### Detection of *cagA*, *vacA* and *dupA* genes

The analysis of the presence of target genes, *cagA*, *dupA and vacA* genotypes was performed through PCR, using one set of oligonucleotides for each gene fragment (Table 1).

Genomic DNA was amplified by PCR for *cagA* according to Rasmussen *et al.*[9], van Doorn *et al.*[14], using a 232-pb fragment. The “s” and “m” regions of *vacA* were genotyped with the previously described primer sets SA/SC and MA/MB. The SA/SC primers amplified “s1” fragments of 176 bp and “s2” fragments of 203 bp. The “m1” fragments were 400 bp and the “m2” fragments were 475 bp [9,13-15] (Table 1).

To evaluate the presence of the *dupA* gene, we used the primers described by Gomes *et al.*[6] which amplifies a 197-bp fragment. It is important to note that these primers were constructed in well-conserved regions based on Brazilian strains.

### Histopathology

Gastric mucosa biopsy specimens were fixed in 10% buffered formalin, embedded in paraffin, sequentially cut and stained with haematoxylin and eosin and Giemsa stain. The histological parameters were graded using the criteria described in the Sydney system for analysis of chronic inflammation, polymorphonuclear activity and intestinal metaplasia [16].

### Statistical analysis

Associations among the virulence factors were evaluated by the two-tailed Chi-square test with Yates’ correction for each group of patients separately. Differences were considered significant when *p* value was less than 0.05. All statistical analyses were performed with SPSS version 20.0.

## Results

Histopathology analyses were performed in 168/205 (82%) patients (99 adults and 69 children) and they revealed 153 patients with chronic gastritis, 13 patients with normal gastric mucosa and just two patients with duodenal ulcer. These results suggest an association between chronic gastritis and the presence of *H. pylori.*

### *cagA*, *vacA* and *dupA* status

Overall, the *cagA* gene was obtained in 98/205 (47.8%) samples. If we considered age, *cagA* gene was detected in 51/105 and 47/100 samples of adults and pediatric patients respectively (Table 2).

**Table 2:** **Frequency of genes *cagA *** **and *dupA *** **and genotypes of *vacA *** **in *H. pylori *** **strains isolated from 105 adults and 100 children with gastric chronic and normal gastric mucosa**

**Genes**	**N (%)**	**Samples from N (%)**		**Histopathology**			
				**Chronic gastritis**		**NGT**	
	***Total***	***Adults***	***Children***	***Adults***	***Children***	***Adults***	***Children***
*cagA* +	98 (47.8)	51 (48.6)	47 (47)	47 (30.7)	26 (16.9)	1 (7.7)	4 (30.75)
*cagA –*	107 (52.2)	54 (51.4)	53 (53)	48 (31.4)	32 (21)	1 (7.7)	7 (53.85)
*dupA +*	85 (41.4)	48 (45.7)	37 (37)	44 (28.7)	26 (16.9)	1 (7.7)	2 (15.4)
*dupA –*	120 (58.6)	57 (54.3)	63 (63)	51 (33.3)	32 (21)	1 (7.7)	9 (69.2)
*s1/m1*	103 (50.2)	58 (55.2)	45 (45)	54 (35.3)	26 (16.9)	2 (15.4)	4 (30.75)
*s2/m2*	75 (36.6)	33 (31.4)	42 (42)	28 (18.4)	26 (16.9)	0 (–)	4 (30.75)
*s1/m2*	17 (8.3)	10 (9.5)	7 (7)	10 (6.5)	4 (2.7)	0 (–)	2 (15.4)
*s2/m1*	1 (0.5)	0 (–)	1 (1)	0 (–)	0 (–)	0 (–)	0 (–)
*s1/s2/m1/m2*	9 (4.4)	4 (3.9)	5 (5)	3 (1.9)	2 (1.4)	0 (–)	1 (7.7)
**Total**	**205 (100)**	**105 (100)**	**100 (100)**	**153 (100)**		**13 (100)**	

NGT: normal gastric tissue.

Regarding the *vacA* gene, the most virulent allelic combination s1/m1 was found in 58 (55.2%) of the *H. pylori* strains, the s2/m2 was detected in 33 (31.4%) and the other genotypes, s1/m2 and s1/s2/m1/m2 were verified in 14 (13.4%) samples from adults. In children, we isolated 45 (45%) strains of the common *vacA* genotype s1/m1 and 42 (42%) strains of *vacA* s2/m2. Thirteen percent (13 strains) had *vacA* genotype s1/m2, s2/m1 and s1/s2/m1/m2 (Table 2).

The *dupA* gene was considered positive when harboring the jhp0917 and jhp0918 genes. *dupA* was detected in 48 (45.7%) and in 37 (37%) *H. pylori* strains from adults and children, respectively (Table 2).

When children and adults were compared, the *dupA* gene was more frequent in strains from adults, but this difference was not statistically significant (p = 0.2610). Similar results were observed for *cagA* gene (p = 0.932) and genotypes of *vacA* (p = 0.1414).

### Association of *dupA* gene with *cagA* and *vacA* genes

When the total gastric samples were collectively analyzed (adults and children), the presence of *dupA* was associated with *cagA* positive strains (p = 0.0003; RR = 1.73; CI = 1.3–2.3). A similar association was verified between the *dupA* gene and the s1/m1 genotypes of *vacA* gene (p = 0.0001; RR = 1.72; CI = 1.3–2.2). As expected, the virulent allelic combination s1/m1 was associated with *cagA* positive (p = 0.0001; RR = 3.52; CI = 2.5–4.99). When children and adults were analyzed separately, a similar association was observed (data not shown).

It is worthwhile to emphasize that the combination of *dupA* positive, *cagA* positive and s1/m1 genotype was found in 47 strains of *H. pylori* suggesting a possible relationship among the main virulence factors.

## Discussion

*H. pylori* has a large degree of genomic and allelic diversity, particularly in the “plasticity region” of the genome, which is composed of the *dupA* gene described by Lu *et al.*[10]. First, *dupA* gene was associated with an increased risk of duodenal ulcer and protection against gastric cancer in Japan and Korea, but these results are controversial and there can be variables depending mainly on the geographic areas studied.

Analyzing the Brazilian population, we detected the *dupA* gene in 85 (41.5%) patients, of which 48 (23.4%) adults and 37 (18.1%) children. Gomes *et al.*[6] found different values and registered the presence of *dupA* gene in 89.46 and 100% of adult and pediatric patients, respectively. Although we employed the primers described by Gomes *et al.*[6], such discordant results may be explained by the different geographic areas of Brazil, studied population, method for molecular analysis and lost or rearrangement in the plasticity zone.

Arachchi *et al.*[17] studying the Indian population, reported the presence of *dupA* in 37.5% of patients with duodenal ulcer (DU) and 22.86% in patients with functional dyspepsia. In addition, Zhang*, et al.*[18] detected *dupA* gene in 35.3% of the Chinese population. Both authors revealed that the prevalence of the *dupA* was significantly higher in strains from duodenal ulcer confirming the original results, which showed that *dupA* was associated with DU and can be a virulence marker to this specific disease. However, the present study reported a similar prevalence of *dupA* in patients with gastritis.

Recently, Imagawa *et al.*[19] showed that patients infected with *dupA*-positive strains have a gastric acid output significantly higher than *dupA*-negative patients. In addition, Abadi *et al.*[20] observed higher acid resistance of the *dupA*-positive strains which suggest that these strains are adapted to a stomach with high gastric acid output. Together, these results may explain the associations between the *dupA* gene and an increased risk for DU formation.

Regarding the detection of *cagA* gene, our results corroborate previous studies in Western countries, with 47.8% of *cagA*-positive patients, without significant differences between adults and children [6,9,15,21,22]. Other interesting result was the association between the presence of *dupA* and *cagA* genes in adults, children and when both groups were analyzed simultaneously. Arachchi *et al.*[17] and Gomes *et al.*[6] also found similar results; however, these authors reported this association only in patients with DU and in adults.

*vacA* genotypes were also investigated in the present study, and an important association was found between s1/m1 genotype and the presence of *dupA* gene, whereas *H. pylori dupA*-negative strains were associated with genotype s2/m2 of *vacA* gene. Similar results were reported by Arachchi *et al.*[17], but with the alleles s1 and m1 separately. Interestingly, strains of *H. pylori* with the combination *dupA* positive, *cagA* positive and s1/m1 were found in 47 (23%) patients. To the best of our knowledge, only Arachchi *et al.*[17] had described the same association, but they found only three patients with functional dyspepsia infected with strains *dupA* positive, *cagA* positive and s1/m1. The present results reveal an important association and suggest a possible relationship among the main virulence factors and the development of severe gastric disease.

Our work has a few limitations limitations. The studied patients had only gastritis and it would be interesting to conduct the same research on patients with duodenal ulcer and gastric cancer. It is important to note that *dupA* gene was detected using PCR methods, showing the presence of genes jhp0917 and jhp0918. Queiroz *et al.*[11] suggest that the presence of jhp0917 and jhp0918 genes may not be enough to characterize an intact and maybe functional *dupA* and that an analysis of *dupA* gene with other molecular biology techniques is important.

## Conclusions

*dupA* is highly frequent in Brazil and it can be considered an important virulence marker. However, further studies are necessary to state that this is a specific-disease marker. In addition, the association among the major virulence markers in adults and children represents new information that can be associated with the prognosis of patients with severe gastric disease.

## Ethics committee approval

Informed consent was obtained from all patients or their legal guardians for publication of the present study. The present study was approved by the Research Ethics Committee of Sacred Heart University (process n. 068/12).

## Competing interests

The authors declare that there are no competing interests.

## Authors’ contributions

WNP, MAF, LMZ and JPBX performed the sample collection, DNA extraction and molecular analysis. RWdeL, WAO and ACN participated in the study design, performed the statistical analysis and provided technical support and scientific discussions. SLMP and LTR conceived the study, participated in its design, coordination and helped to draft the manuscript. All authors read and approved the final manuscript.
